# Words Left Unspoken: Exploring the Unspoken Narratives of Loved Ones Supporting a Person who has Survived a Suicide Attempt

**DOI:** 10.1177/00302228251328422

**Published:** 2025-04-14

**Authors:** Lauren Brockett, Katie High, Farah Dauhoo, Jennifer Heath

**Affiliations:** 1 Doctorate Programme in Clinical Psychology, School of Health, Medicine and Life Sciences, College Lane Campus, 3769 University of Hertfordshire, Hatfield, UK; 2 4955 East London NHS Foundation Trust, Child & Adolescent Mental Health Service, Looked After Children’s Team, Bedford, UK

**Keywords:** suicide, informal care, attempted suicide, loved-one, unspoken narrative, carer support

## Abstract

Experiences of informal carers supporting a loved one following a suicide attempt are under-reported in research. This is partly due to specific psychological barriers which lead to carers feeling silenced. This study aimed to uncover and explore unspoken narratives of informal carers who have provided care for a loved one following a suicide attempt. Fourteen adult participants responded to a social media advert inviting them to an interview to talk about their experience of caregiving. Interviews were analysed using Reflective Thematic Analysis. Five main themes were identified: ‘The Conspiracy of Silence’, Unequivocally Unspoken’, ‘A Sense of Responsibility’, ‘Making Comparisons’, and ‘Relationships with Professionals’. Results are discussed in relation to existing literature before highlighting clinical implications. Researchers and clinicians should aspire to learn more about the experience of informal caregivers in this context, aiming for carers to be able to access appropriate service provision and receive the care and support they deserve.

## Introduction

When an individual makes a suicide attempt there are two possible outcomes: they die, or they survive. Research into suicide bereavement is limited. That which exists indicates that suicide bereavement is different to being bereaved through a more ‘normative’ mode of death (Jordan, 2020). Literature often describes those who have been bereaved by suicide as ‘suicide survivors’ (Jordan & McIntosh, 2011); a phrase with connotations which are not typically seen in bereavement research more widely. Suicide survivors are likely to experience a complex grief reaction (Ali, 2015), and have a higher chance of experiencing Post Traumatic Stress Disorder (PTSD; Mitchell & Terhorst, 2017), especially if they were the one to find the deceased individual (Jordan, 2020).

Clinicians working in suicide bereavement have found that suicide survivors feel an increased sense of guilt and responsibility for the deceased, they experience heightened stigma and shame (Geležėlytė et al., 2020), express an increased desire to make sense of what happened, are more likely to experience social isolation, and in many cases, suicide survivors will experience their own suicidal thoughts (Jordan, 2020).

When an individual makes a suicide attempt and doesn’t die, family and friends (those who would fall into the “suicide bereaved” category) are still impacted. Yet, research into the notion of being almost bereaved by suicide is more-or-less non-existent. Suicide research focuses predominantly on the individual deemed as ‘suicidal’, with little academic thought given to their loved ones. In the instance of a suicide death, academic interest shifts towards the experience of loved ones; but in the instance of an *almost* suicide death, interest remains solely focused on the suicidal individual.

The experience of informal carers supporting a loved one following a suicide attempt is near non-existent in academic literature. This may be in part due to psychological barriers, such as self-blame (Daly, 2005), shame (Asare-Doku et al., 2017), and a lack of trust in others (Dempsey et al., 2019), which leads to carers feeling silenced, and as if they cannot share their stories openly. This research was developed holding this in mind, and a specific focus was put onto what has been unreported in literature so far. Consequently, this study focuses on the unspoken narratives of informal caregivers supporting somebody following a suicide attempt. A specific focus on the unspoken was developed with the aim of opening up conversations hidden by the aforementioned psychological barriers (Asare-Doku et al., 2017; Daly, 2005; Dempsey et al., 2019).

## Method

This research received favourable ethical opinion from the University of Hertfordshire Research Ethics Committee (protocol number: LMS/PGR/UH/05362).

### Design

As the research seeks an understanding of the human experience, a qualitative study was designed (Austin & Sutton, 2014). The method leans into the idea of ‘intersubjectivity’, a concept encompassing the way individuals construct and attach meaning to specific social or human problems (Creswell, 2014; Todd et al., 2004). Qualitative research has a rich history in advancing the study of human behaviour (Agius, 2013); and as such, it has been argued that qualitative research is *“essential”* in developing the field of suicidology/suicidological research (Hjelmeland & Knizek, 2010). The phenomenological approach encourages exploration into the intricacies and nuances that are simply being human, whilst respecting and valuing individual difference (Hedge, 2021).

### Researcher Positionality

The researchers’ theoretical lens informs how decisions are made in relation to the research, and how the methodology is executed. It is underpinned by the researchers ontological and epistemological positionality. This research was conducted from the theoretical perspective of Critical Realism (CR). Through the lens of CR, knowledge of social phenomena is understood to be subjective, relative and socially constructed by individuals; it is acknowledged that a reality exists outside of ones knowledge of it (Stutchbury, 2022), and that this reality cannot be unproblematically understood (Wynn & Williams, 2012). Applying CR to this research provides a framework to understand the experiences of participants. It encourages the researcher to be critical of the narratives provided, understanding that participants’ narratives are personal to them and their specific context.

The personal positionality of the first author, who collected and analysed the data, was continually considered; and reflexivity (through bracketing, reflective journalling, and supervision meetings) was practiced throughout the research journey.

### Recruitment

It was hypothesised that recruitment to this research could be challenging due to the area of interest being sensitive and unspoken. A detailed recruitment plan was developed to assist in considering and overcoming challenges (Kristensen & Ravn, 2015).

#### Recruitment Strategy

Step 1: The first step in a successful recruitment strategy is to become familiar with the population of interest (Coyne et al., 2019). Local and national charities and services, supporting individuals who have been affected by suicide, were identified via Internet searches. Several charities and other relevant professionals (e.g., local authority suicide prevention leads) were contacted. Initial contact messages shared information about the research, and invited individuals to get in contact with the first author via email if they wanted more information. Sixteen responses were received, and network meetings were scheduled. Seven individuals offered their services in a consultation capacity. These individuals formed an advisory panel, and consultation was invited and encouraged at every stage of the research process.

Step 2: The advisory panel reviewed draft versions of the research advertisement poster, consent form, participant information form, debrief form, and interview schedule. Changes were made in line with the recommendations made. All consultation occurred via the Microsoft video conferencing platform, MSTeams.

Step 3: Poster advertisements were sent to relevant charities/services for sharing on their online platforms to attract participants. The sampling techniques used were convenience sampling and snowball sampling.

### Participants

Participants expressed interest in the research by emailing the first author after viewing the advertisement poster. Once expression of interest was received, the first author sent the participant information form and asked potential participants to review this and get back in contact should they wish to participate. The participant information form detailed the inclusion and exclusion criteria (Table 1), and participants were asked to check their eligibility against this. All participants who agreed to take part in an interview were sent a copy of the interview schedule so that they knew what to expect on the day. They were also sent the consent form and asked to return this prior to the interview date.

**Table 1.** Participant Inclusion/Exclusion Criteria.

Participant inclusion criteria	Participant exclusion criteria
• To be over the age of 18	• The ‘loved one’ has made multiple suicide attempts
• To be a resident in the UK	• The participant, or their loved one, are personally known to the researcher
• To be English speaking	
• To have experience of supporting someone, a ‘loved one’, following a suicide attempt	
• The suicide attempt must have taken place more than six months ago (this allows for an extended period of ‘watchful waiting’; NICE, 2018)	
• The participant must have had the belief that their loved one’s intention was to die	

### Data Collection

Twelve interviews took place online, via MSTeams. Two interviews were conducted via telephone. All 14 interviews were recorded using MSTeams. For the two interviews which were conducted over the phone, MSTeams was used to record the audio with the participants’ consent.

#### Interview Schedule

The semi-structured interview schedule was developed in conjunction with the second author, and reviewed by the advisory panel. The interview schedule was broken down into four sections: (1) relationship to person, (2) experience of suicide attempt, (3) unspoken narratives, and (4) support.

#### Interview Procedure

Prior to the recorded interview, introductions were made, and participants were given the opportunity to ask any questions or share any worries/anxieties they had. Their completed consent form was reviewed to ensure everything had been understood and fully informed consent was given for participation. Anonymity, confidentiality, and the participant’s right to withdraw were re-explained to the participant. The recording was started, and participant demographic information was taken before formally starting the interview.

Before closing the interview, the first author checked all questions had been asked, and gave the participant space to offer anything they felt was relevant but had not disclosed.

### Data Analysis

Transcripts were anonymised and analysed using Reflexive Thematic Analysis (RTA; Braun & Clarke, 2006, 2013, 2019) using NVivo 14 (QSR International, 2023). The data analysis was carried out in accordance with the six-step framework set out for effective RTA (Braun & Clarke, 2013).

## Results

Participant demographic information, as well as details of their relationship to their ‘loved one’, are presented in Table 2.

**Table 2.** Participant Demographic Information.

Pseudonym	Age	Gender	Ethnicity	Geographical location (within the UK)	“The loved one was my…”	Approx. How long ago did the experience occur?
Abigail	52	Female	White caucasian	South-East	Uncle	20 years
Elizabeth	49	Female	White British	South-East	Son	7 years
Harry	60	Male	White Irish	South-East	Friend	1 year
Jane	50	Female	White British	Midlands	Daughter	4 years
Noah	52	Male	White British	South-East	Girlfriend	10 years
Alice	65	Female	White British	North-West	Sister	29 years
Camila	48	Female	White European	East Midlands	Daughter	8 years
Harper	26	Female	White British	South-East	Brother	7 years
Ruby	57	Female	White British	East	Son	2 years
Grace	55	Female	White Irish	South-West	Daughter	6 years
Daniel	41	Male	White British	Midlands	Friend	15 years
Quinn	53	Female	White British	North	Husband	5 years
Sofia	77	Female	White British	North-East	Son	25 years
Evie	60	Female	White British	South-East	Son	5 years

Five themes and eight subthemes were developed from the data using RTA (Braun & Clarke, 2013). These are illustrated in Table 3.

**Table 3.** Themes and Subthemes.

Theme	Subtheme
The conspiracy of silence	“It’s their story to tell”
	Myriad of protection
	Systemic influences
Unequivocally unspoken	Bearing Witness
	“Skirt [ing] around it”
	Overwhelming emotions
A sense of responsibility	
Making comparisons	
Relationships with professionals	Positive experiences
	Spoken but unheard

Each theme is described with corresponding quotes. Where the method of suicide attempt was mentioned in the quotes used, detail has been removed to protect the vulnerability of the person, and the wishes of the participants.

### Theme 1: The Conspiracy of Silence

This theme captures competing ideas, concepts and influences that participants felt contributed to their experiences being unspoken.

“...it’s like a conspiracy of silence, I am being silenced.” (Evie, mother)

#### Subtheme 1: “It’s [Their] Story to Tell”

Unspoken narratives around story ownership, and subsequent feelings of powerlessness, are explored in this subtheme. Participants universally described how they felt they did not have ownership of the experiences that had involved them stepping into the carer role and acting in a carer capacity. Participants believed that the experiences belonged to the person who made the suicide attempt, and thus did not feel as though they had the right to share any of what they had experienced with others.

“I kept it to myself. I kept it in really, to be honest. [...] I was probably... I feel like it’s not my place to say anything. That’s why, yeah. It’s her journey and it’s her story to tell. It’s not mine as such.” (Daniel, friend)

Not having ownership/control over their experiences could leave participants feeling powerless. Powerlessness was evidenced when some participants described how their loved one asked them to share falsehoods to hide the suicide attempt. The belief that this was their loved one’s story, meant that participants felt obliged to disseminate it in a way their loved one saw fit.

“[My daughter] didn’t want people to know. So, we had... Obviously, once she got through it and we found out she was ok, you know, physically. We had to... Well, I had to concoct a make-believe story of why she was admitted, and why she was in high dependency for so long and everything else.” (Camila, mother)

#### Subtheme 2: Myriad of Protection

Each participant described a distinct need to protect either themselves, their loved one, and/or people they are in conversation with, from distress that might be caused from sharing experiences related to caregiving (e.g., details of the suicide attempt).

Participants collectively explained how they chose not to speak about their experiences directly with their loved one, believing that doing so would cause their loved one untoward distress.

“And I didn’t want to bring it up because I didn’t want to cause him pain.” (Abigail, niece)

Desire to protect their loved one from the potentially problematic views of others led to further silencing of their experiences.

“I just felt I couldn’t really talk much about it because she knew a lot of our friends. [...] I didn’t want them to see her differently. [...] I was trying to protect her from what other people might think of her.” (Alice, sibling)

Carers explained how a desire to protect themselves from anticipated unkind responses also stopped them from talking to others.

“And explaining that to an outsider is... People’s normal reaction is ‘well, why are you still there?’. You know, ‘why haven’t why haven’t you run away from this situation?’ And that wouldn’t have been helpful to me.” (Sofia, mother)

Almost all participants described a desire to protect the person receiving the information from experiencing distressing emotions as motivation for not sharing their experiences.

“It was more protection for other people. They don’t need that image.” (Jane, mother)

#### Subtheme 3: Systemic Influences

Participants outlined several systemic influences causing them to feel as though they could not share their experiences with others. All participants alluded to how an awareness of wider societal norms, and societal perceptions of suicide/suicide attempts, ensured that their narratives remained unspoken.

“Society’s judgments as well and stuff like that. It makes you feel... It’s like the peer pressure, type thing. Like you shouldn’t talk about it. It’s like the taboo in the room.” (Daniel, friend)

British societal and cultural norms appeared to act alongside generational norms. As well as participants feeling as though an appropriate context did not present itself for them to share their stories, they also seemed to feel as though generational norms dictated who it was, and was not, appropriate to share experiences of suicide/suicide attempts with.

“My dad is like the ultimate stereotypical male of that generation [...] could not talk about any of it. [...] We just can’t go there so with him, like he was definitely someone that was like, it was just kind of, out of, almost out of bounds.” (Harper, sibling)

For participants who were supporting their loved one through the pandemic, the social distancing rules and restrictions imposed by the UK Government acted as another method of systemic silencing.

“I wasn’t socialising in the way I would have done before Covid. [...] We weren’t living our normal lives. We were all living, you know, at home. We weren’t going out, you know, to suppers or whatever. All those normal activities where you would say, ‘oh, my son’s not well.” (Ruby, mother)

All of the above contribute to stigma existing around suicide. Participants described how the stigma attached to suicide led to them feeling as though they could not share their stories with others as people would not want to know.

“People say there’s no stigma now around mental health. That’s rubbish. It’s a fear of the unknown. People don’t want to know what they can’t see.” (Sofia, mother)

### Theme 2: Unequivocally Unspoken

This theme details the parts of the carers’ stories that are clearly, and unambiguously, unspoken.

#### Subtheme 1: Bearing Witness

This subtheme refers to the suicide attempt itself. For some participants, this is what they actually saw; for others who were not present at the time of the attempt, it includes the intimate details of what they know to have happened.

Details of method were described as unspoken due to carers being aware of just how vulnerable their loved one was in the moment.

“I didn’t talk about method with people. [...] I wouldn’t have gone into detail about what she’d done [...] I don’t think that’s anybody’s business.” (Jane, mother)

In three of the 14 interviews, detail regarding method was not discussed at all. This ensured the narratives remained unspoken, even with the researcher explicitly inviting discussion of the unspoken narratives.

When discussing why it was so difficult to talk about method, participants reflected on how talking about the realities of what happened impacted them.

“I don’t wanna come back to it. Don’t wanna keep going over it because every time you tell someone else, it brings it all up again.” (Noah, partner)

Initially, participants explained how the details of method had never been shared with another person. However, throughout each interview it became clear that this was not the case. For almost all participants, it became unspoken over time. At the point the suicide attempt occurred, when shock and adrenaline took over, nothing was unspoken.

#### Subtheme 2: “Skirt [ing] Around It”

Some participants were observed not to use the word ‘suicide’ in conversation with the researcher, or when they were describing interactions with others, throughout the entire interview. When answering questions about their experience, these participants moved through the story with a pause where one would assume a reference to the suicide attempt might be made.

“That led to him to, sadly... [pause] Because they’ve been together 50 years...” (Abigail, niece)

“He... [pause] So, it happened when he was at Uni. He... [pause] I guess he always struggled with his mental health.” (Harper, sibling)

Some participants described how the word suicide was unspoken between them and their loved one, with the participant choosing to “*skirt around it*” and find other language as a result.

“I bought it up on/at/on probably about two or three occasions, and I sort of said, and I’d sort of say, ‘oh, about that [reference to method] situation’. I’d kind of sort of skirt around it like that” (Grace, mother)

#### Subtheme 3: Overwhelming Emotions

Participants described how the personal story of their emotions/internal psychological processes were unspoken out of choice at the point the suicide attempt took place. Carers described how they did not feel they could cope with allowing their emotions to have space in the period of time following the suicide attempt.

“[There was a] barricade to accessing the emotional, as such. I was too busy on guard to be able to connect it too much emotionally really. So yes, it was like robot.” (Daniel, friend)

When describing the feelings being hidden, many of the participants expressed self-blame for the suicide attempt taking place.

“All I wanted for any of my children was to grow up and be happy. [...] I absolutely blame myself for that not happening” (Grace, mother)

Developing these feelings further, for mothers, self-blame could move through guilt and into shame.

“You feel like a failure is as a parent because you should have noticed. And why, why, why has it got to a place where your child is so vulnerable? Surely you failed as a parent. Your whole job is to look after them.” (Evie, mother)

A felt and present (in some cases) sense of fear was active in all interviews. Participants described being fearful of almost every interaction they had with their loved one, worrying they would say something, which would make things worse, and another suicide attempt would occur.

“The commonest fear that I had is saying the wrong thing and making the situation worse.” (Harry, friend)

### Theme 3: A Sense of Responsibility

This theme captures an unspoken, felt sense of responsibility experienced by carers.

Mothers connected intensely with responsibility. Some reflected on their lives to find evidence positioning them as responsible for their child’s suicide attempt.

“And that’s why I blame myself... She had a history of seeing me do it. You know? So, it’s like, not... I mean, I’ve never tried to [reference to method], but I have a history of [reference to method].” (Grace, mother)

Almost all carers felt responsible for ensuring that their loved one felt better.

“I must have affected his mental health. So, I was/as soon as he was starting to have mental health problems, I blamed myself. And so, when it came to him saying this, [...] I thought, ‘right I must. I must deal with this’. I/this is, you know. ‘I/it’s up to me’.” (Evie, mother)

This took the form of checking in on their loved one practically, and ensuring that the environment was ‘safe’.

“I do remember getting home from the hospital and then sort of almost locking up the medicine cabinet and, you know, getting rid of stuff and, moving things away that thought I thought might be, you know, triggering.” (Elizabeth, mother)

Or noting changes in the persons mood and mental state to try to keep an eye on what was going on for them.

“I’d been writing notes, and I’ve got the booklets where I was writing it down so that we could monitor his, you know, changes and how he’s feeling.” (Quinn, wife)

### Theme 4: Making Comparisons

This theme highlights an unspoken sense of unfairness felt by carers. The unspoken narratives in this subtheme are expressed through carers making comparisons to physical illness, and bereavement.

Some participants compared suicidality to cancer. Camila tracked her daughter’s journey throughout the health system according to cancer stages. Using this analogy, she concluded that, in the case of mental health, it often is too late for treatment to work when care is finally provided.

“I do describe it as cancer that, you know, by the time [my daughter] actually saw somebody it [had] already been six months, she’d already reached Stage 1. By the time she sees someone else, and she's waited its Stage 2, Stage 2/Stage 3. By time the she finally got help [...] it was Stage 4, it was terminal. (Camila, mother)

When participants compared themselves to someone who has been bereaved by suicide, some participants reflected on how their experiences felt less than by comparison.

“...I kind of/I then don’t necessarily open up. You know, if they are sharing their loss with me, I don’t then necessarily open up to say I’ve been through something similar because, because I’m not experiencing what they’re experiencing, if that makes sense, and it’s not fair for me to share that...” (Elizabeth, mother)

### Theme 5: Relationships with Professionals

This theme explores the unspoken ups and downs/inconsistencies in the relationship between carers and professionals. The term ‘professionals’ used here extends beyond healthcare professionals, including anyone with whom the carer interacted in a professional capacity.

#### Subtheme 1: Positive Experiences

The way in which professionals were observed to interact with their loved one was a distinguishing factor as to whether carers deemed the experience to be positive. Professionals who were observed to provide care in a compassionate way were valued by participants. The fact that participants could make these observations suggests that they were included in the care provided, which is possibly another factor contributing to a more positive experience.

“The actual physical health nurse who [reference to method] was amazing. Actually, couldn’t have faulted her. Was very sympathetic, understanding. A bit mummy-ish like that...” (Jane, mother)

Having their position as a carer recognised was relevant in all reports of helpful/positive interactions with professionals.

“There was a wonderful nurse on the ward […] she asked me lots of questions and she was literally just as a junior nurse. She wasn’t any kind of psychologist or anything. But actually, it was really helpful having her kind of, give me the space to talk.” (Quinn, wife)

#### Subtheme 2: Spoken but Unheard

Carers discussed times where they had spoken directly to professionals, expecting to be listened to, but were not (or, at least, felt they were not).

Many participants voiced the severity of their loved one’s suicide attempt to professionals but felt as though the severity was not heard/taken seriously.

“The [paramedic] said, ‘well, if [your son] wants to jump out of the window, just let him’... Wasn’t that an extraordinary thing to say!” (Ruby, mother)

This led to participants feeling disillusioned by professionals, and indirectly led to them feeling as though they had to take on more of a caregiving role as they did not feel supported.

Some participants submitted complaints to professional services about the service they had received. It seemed that, even when carers spoke their stories in this capacity, they were still not heard/listened to in a way that felt acceptable.

“I made a formal complaint in November. It took them six months to answer that. Then they sent a one-page document which was very flippant, dismissive. Saying that all the questions that I have wanted answered had already been answered...” (Noah, partner)

## Discussion

### Story Ownership, Protection and Systemic Influences

The medical model positions suicide as a symptom of mental health difficulties. Mental health stigma is well studied in literature. Modified Labelling Theory (Link et al., 1989) is a framework that can be used to help understand some of the experiences described by participants. The model posits that individuals deemed to be ‘unwell’ are aware of the stigma associated with mental illness, and implement management strategies to cope with the possible discrimination and social rejection that can occur as a result (Ray & Dollar, 2014). In the subtheme*: “It’s [their] Story to Tell”,* participants described feeling as though they had to make up stories, as directed by their loved one, to cover up the fact a suicide attempt had been made. This fits with one of the key ‘coping strategies’ in Link’s (1989) theory, which is for the person deemed unwell to conceal their mental illness to avoid further labelling and stigma. By sharing false narratives, participants help their loved ones avoid stigma but inadvertently leave themselves unable to seek/access support for their own emotional responses to the upsetting event evoking feelings of powerlessness.

A carers desire to protect the care receiver was observed widely as a theme throughout broader carer research. For example, in Boudioni et al.’s (2015) study, participants were observed to be protecting their loved one when interacting with the researcher through *“constant touching, eye contact, or leaning their body toward them”* (p. 535). This example, and the examples discussed by participants in this study (evidenced in subtheme: *Myriad of Protection*) likely links with societal norms around what it means to be a carer, and the subsequent identity that carers resume when they step into the role.

Participants described how an awareness of British societal and cultural norms meant that they felt they could not share their experiences openly. Phenomena such as this have been explained in literature using the Spiral of Silence model (Noelle-Neumann, 1974). The model dictates that, as human beings, we have a natural fear of social isolation, which prompts us to constantly monitor for signs of approval or disapproval. To avoid disapproval, we refrain from talking about things perceived to be controversial, such as suicide. A recent systematic review has suggested that fears around disapproval permeate into academia, providing a challenge for many researchers hoping to complete suicide-related research. High’s (2024) review indicated that societal and cultural norms surrounding suicide can influence how straightforward the process of gaining ethical approval can be when proposing such research. These findings underscore the powerful role societal norms play in shaping both personal disclosure, and academic inquiry in this area.

### Language

Participants in this study were observed to have difficulties interacting with the word “suicide”: some participants avoided the word completely; others used other words to verbalise the event; and others were able to reflect on how their interactions with the word suicide had changed over time. Avoidance of the word can be explained through the Modified Labelling Theory (Link et al., 1989), mentioned earlier. This model promotes ideas around *not* using labels as a means of actively reducing associated stigma.

In recent years, anti-stigma movements and language campaigns, headed by charities such as Samaritans, have offered alternative ways to consider language use. Interestingly, these campaigns support movements away from words that prefix suicide, as opposed to moving away from using the term suicide itself. Anti-stigma campaigns have advocated to move away from “committed” suicide due to the criminal connotations, and also to move away from “completed/non- completed” suicide due to connotations around achievement. These campaigns are supported by research, such as that by Padmanathan et al. (2019), who surveyed 3000 people affected by suicide and found that *“attempted suicide,” “took their own life,” “died by suicide,”* and *“ended their life”* were preferred terms/phraseology. The advice from anti-stigma campaigns and research does not fit with what was observed of participants, who were often opting to not use the word at all.

The social media platform TikTok recently shared data revealing that the term suicide was not being used as commonly as it once was, the preferred replacement was “unalive”. The hashtag #unalivemeplease has 9.2 million views on TikTok; #unaliving has 6.6 million; #unaliveawareness has an additional 2.2 million. The hashtag #suicideprevention is a frequently used tag on the app, whereas the hashtags #suicide and #suicideawareness do not exist (Tait, 2022). This suggests that similar to the participants in the sample, there is a desire to move away from the word suicide. However, concerns have been raised regarding changing to a word such as “unalive”. Deborah Tannen argues that “*coming up with alternate or roundabout ways to say things, in order to avoid saying them outright, sends a message that the meaning is unsayable”* (Tait, 2022). Ultimately, a move away from the word could uphold the silence through promoting avoidance; perhaps work would be better spent on anti-stigma campaigns, research, and education so that the word itself does not hold so much fear and power.

### The Role of a Carer

Participants described how they connected with an unspoken sense of responsibility when they stepped into the carer role. In many ways, the sense of responsibility links with the idea of *Role Engulfment* (Eifert et al., 2015). *Role Engulfment* occurs as the carer takes on more and more responsibilities to ensure the health of their loved one, to the detriment of their own identity. Oudijk et al. (2011) found that, in a sample of physical health related family carers, the sense of responsibility was linked with a sense of obligation, and this obligation made participants more inclined to provide care. The authors talk about a “trade-off” relationship between carers and their loved ones, that in many ways carers are “repaying” loved ones for help received in the past; or that this is simply a reflection of traditional social norms around what it means to care for someone.

Participants made comparisons between their position as carers for somebody with suicidality, and other phenomena. Social Comparison Theory (Festinger, 1954; Wood, 1989) asserts that people assess their self-worth by making comparisons to others. Festinger (1954) proposed *upwards social comparison* as the comparison between us and those we perceive as better off than us. The model dictates that initially, with upwards social comparison, we might feel discouraged but eventually this can work as motivation to get to the place where we perceive the other person to be. For the participants in the sample, many of them are now working in the suicide prevention space. This could be explained as them trying to change to ensure other people have access to better services that they didn’t have, spurred on by upwards social comparison.

There is a lack of service provision available to support these carers in the UK. Current service provision does not include support spaces (e.g., peer support) for informal carers (NHS England, 2016, 2019), meaning that there is no space provided for carers to meet, feel connected, and rationalise their thoughts around comparison with others. Therefore, comparison with others, particularly those accessing more adequate support as carers, may be another explanation as to why many participants in this sample now work in suicide prevention.

### Professional Engagement

Participants described the way in which they interacted with professionals to be an important part of their narratives. Positive experiences occurred when professionals took time to understand the complexity of suicidality for both them and their loved one. Trauma-informed practice is a movement gaining traction across healthcare services. Trauma-informed approaches are based on promoting the principals of safety, trustworthiness, collaboration, empowerment, and choice (Harris & Fallot, 2001) when interacting with others. It is an approach that fits with the description of the positive experiences of professional engagement expressed by participants.

Negative carer experiences are not uncommon in literature. A reflective article from a carer for someone with mental illness shared: *“From my experience, carers voices may at times be the loudest in the room but yet remain unheard”* (Daniels, 2023, p. 1784). The author ponders whether it is of benefit to professionals, in a context of overstretched services due to austerity, to keep carers voices unspoken as the non-acknowledgment of carers increases the responsibility placed on carers and decreases that on professionals. Daniels (2023) also comments on how legislation to protect carers’ voices is inaccessible due to the jargon used, rendering it ineffective if carers were to try to use it in support of themselves or other carers. Similar to the participants in this sample, Daniels (2023) asks for a seat at the MDT table, to be part of discussions, and to be listened to.

### Strengths and Limitations

#### Strengths

Early consultation and consistent reflexivity throughout this research are strengths. Consultation and reflexivity encouraged consideration of personal biases throughout the research process, and offered regular check-ins with the lived experience population to ensure the research effectively met the needs of the target population.

The research was conducted from the epistemological position of critical realism. This provided a framework for the development of themes across the dataset, obtaining a collective understanding of how participants experienced the aftermath of a suicide attempt (Stutchbury, 2022), whilst simultaneously encouraging critique of the narratives provided, understanding that each narrative has been personally constructed through engagement with different contexts (Wynn & Williams, 2012). This approach meant that, through the findings, overarching themes could be presented, whilst respecting the individual participant stories in the process.

#### Limitations

There was a gender bias in the sample of participants interviewed for the study, which is a sampling limitation. Eleven of the 14 participants were female (78.6%), and three were male (21.4%). Gender bias in this study means that the findings are not fully applicable to the wider population as they do not fairly capture the experiences of the people affected. Further, the sample was made up of entirely White participants. This presents a further sampling limitation with further impacts on the generalisability of the results.

The study did not control for the age of the person who had made the suicide attempt. The age of the person receiving care ranged from age 14 years upwards, meaning the way participants interacted with the caregiving role was varied. For some participants, the responsibility they felt linked not only to their carer identity but to the legal responsibility they held as a parent to a young person under the age of 18 (UK Government, n.d). Such contextual influencers could be viewed as a limitation to the transferability of findings, and could be mitigated in future research by controlling for this information.

The aim of this research was to explore what goes unspoken for caregivers supporting somebody who has survived a suicide attempt. A limitation of any research exploring the unspoken is that much of the unspoken will remain that way. The unspoken narratives that are shared through research and other forums (e.g., peer support spaces), may not be the true unspoken narratives. It is plausible to assume that there is a set of unspoken narratives that feel un-sharable and will never be uncovered. Future research might consider the use of alternative research methods to assist in breaking down barriers that keep stories unspoken [for example, photovoice which is considered a social justice tool, empowering individuals to express difficult stories through art (Budig et al., 2018)].

### Clinical Implications and Invitations for Future Research

#### Clinical Implications

The results of this study demonstrate that carers are very much present and involved in the care of their loved one; often to their own detriment. Healthcare professionals should work towards thinking more systemically to consider the role of the carer in treatment planning, making use of statutory carers assessments whenever a ‘patient’ appears to have family/friends involved in their care.

Professional healthcare services need to consider the trauma that carers experience as part of their role. Therefore, any service that is set up should be done so through a trauma-informed lens. The findings of this study have shown how, when treated with a trauma-informed approach, participants felt their relationships with professionals were more positive.

The development of services/support provisions for carers supporting somebody who has made a suicide attempt is of the upmost importance, requiring urgent thinking in the suicide prevention space. A space should be developed whereby carers feel they can take control of their narratives, and share their experiences in safe and containing spaces. The first step to achieving this could be to implement peer support groups for carers supporting loved ones who have made a suicide attempt. Peer support also has wider recognition for being a great support for carers in other areas, such as family carers for people with dementia (Smith et al., 2018).

#### Invitations for Future Research

This research has specifically looked at the unspoken narratives of caregivers supporting somebody who has survived a suicide attempt. Due to the limited evidence base at present, any future research building on the current understanding of the carer experience will be valuable. Future research should hold the sampling limitations outlined above in mind when approaching new research questions, considering the impact of Social Graces (Burnham, 2012) on how one interacts with the carer role.

The feeling of powerlessness for carers identified in this study has also appeared as a theme in other relevant research (e.g., Boudioni et al., 2015). Understanding more about how a feeling of powerlessness operates, or not, for carers would be incredibly valuable in helping clinicians develop appropriate support packages. Additionally, research looking at the positive experiences of caregiving would also be valued as taking a strengths-based approach could help in peer-support development (Heath, 2020).

To understand more about the systems around carers, research investigating the clinician perspective of carers, or the care receiver’s perspective would also be beneficial.

## Conclusion

The aim of this research was to decipher and explore unspoken narratives that exist for informal caregivers who have supported a loved one following a suicide attempt. Through 14 interviews, numerous unspoken narratives were identified. Participants discussed: Silencing, narratives which are unequivocally unspoken, the responsibility that comes with being a carer, a sense of unfairness, and carers’ turbulent relationship with professionals. Findings are discussed in relation to existing literature to build on the developing evidence base, amplifying the voice of a population who have not previously been given space in academic literature. It is hoped that academics, clinicians, and researchers begin to recognise this population, and learn more about informal caregiver experiences in this context so that, one day soon, they are given the space and time in service provision they so desperately deserve.

## Data Availability

The participants of this study did not give written consent for their data to be shared publicly. Due to this, and the sensitive nature of the research, supporting data is not available.
